# A Case Series on the COVID-19 Vaccines and Possible Immune-Related Adverse Events: A New Challenge for the Rheumatologists

**DOI:** 10.7759/cureus.29660

**Published:** 2022-09-27

**Authors:** Vicky Nahra, Mahesha Makandura, Donald D Anthony, Maya Mattar

**Affiliations:** 1 Rheumatology, University Hospitals Cleveland Medical Center, Cleveland, USA; 2 Immunology, Case Western Reserve University, Cleveland, USA; 3 Rheumatology, Veterans Affairs Medical Center, Cleveland, USA

**Keywords:** case series, inflammatory arthritis, rheumatoid arthritis, vaccines, covid-19

## Abstract

The COVID-19 pandemic has been a prime health issue since December 2019. Consequently, there has been an urgent need to prevent severe acute respiratory syndrome-coronavirus 2 (SARS-CoV-2) infection and its associated morbidity and mortality. The currently available vaccines are designed to prevent infection. Their efficacy and safety have been demonstrated in clinical trials. Yet, given the short duration of the trials and the urgency to start vaccination, adverse events have been reported worldwide in real-life data format. Immune-mediated disease flares or new-onset inflammatory diseases following vaccine administration have recently been reported worldwide. Here, we present three cases of inflammatory arthritis (IA) caused by the BNT162b2 COVID vaccination, including two new-onset cases and one case of a flare of existing disease. The first case is new-onset IA, the second case is new-onset rheumatoid arthritis, and the third case is a flare of existing rheumatoid arthritis.

Given the timeline of when our patients developed either a flare of their existing rheumatoid arthritis or new-onset IA or polymyalgia rheumatica (PMR) (a few days after receiving the COVID-19 vaccine), in addition to the currently available evidence of documented similar cases post administration of mRNA vaccines, as well as the link between their mechanism of action and the pathogenesis of those diseases, we can speculate a causal relationship between the vaccine and the triggering of these disease entities. In the future, it is important to consider that autoimmune diseases might be triggered or flared by the administration of vaccines, which appears to be associated with the COVID vaccine as well. Further evaluation of its incidence will provide additional clarity, though the rarity of this occurrence in the setting of more than half of the US population becoming vaccinated indicates that the benefit of the vaccine in terms of protection from COVID morbidity and mortality far outweighs this risk.

## Introduction

The COVID-19 pandemic has been a prime health issue since December 2019. A cornerstone in the fight against this disease has been the urgent need to prevent severe acute respiratory syndrome-coronavirus 2 (SARS-CoV-2) infection and its associated morbidity and mortality. Among the currently available vaccines designed to prevent infection, tozinameran (BNT162b2), BioNTech-Pfizer, is a nucleoside-modified messenger RNA (mRNA) vaccine encoding the spike (S) protein for the SARS-CoV-2 virus [1]. Its efficacy and safety have been demonstrated in clinical trials. Yet, given the short duration of the trials and the urgency to start vaccination, many adverse events have also been reported worldwide in real-life data format. Immune-mediated disease flares or new-onset inflammatory diseases following vaccine administration have recently been reported worldwide (USA, UK, Italy, Israel, etc.). Following mRNA vaccines, there have been reports of new polymyalgia rheumatica (PMR), rheumatoid arthritis (RA), or inflammatory arthritis (IA) onsets as well as flares of pre-existing autoimmune rheumatic disease. Here, we present three cases of IA flare following the BNT162b2 vaccine, which is a newly developed vaccine and has only been available to the public for a year and a half now, making our case series valuable to the newly available literature and aids in the management of these patients.

Our study did not require ethical board approval because it is a case series that includes only three patients. It is a descriptive paper on autoimmune manifestations post-COVID vaccination. Formal written consent was obtained from all patients to publish their medical information.

## Case presentation

Case 1: COVID vaccine-associated temporary new-onset inflammatory arthritis

A 71-year-old Caucasian male with a history of stroke, brain aneurysm, seizures, and dyslipidemia presented to South West Hospital with the chief complaint of generalized weakness and diffuse joint pain that started one day after his second dose of BioNTech-Pfizer COVID-19 vaccine and has been ongoing for 10 days. Symptoms were progressively getting worse to the point that he was unable to walk. The patient denied any previous history of arthritis, uveitis, sexually transmitted infections, heel pain, or psoriasis. On physical exam, there was no active synovitis, and the neurological exam was normal including muscle tone and power. At that time, the differential diagnosis included an infectious versus noninfectious inflammatory process status post-COVID-19 vaccine. He was evaluated by infectious disease, neurology, and rheumatology during his hospital stay. Infectious work-up included laboratories (complete blood count or CBC, blood culture, chest x-ray, urine analysis, and COVID-19 PCR testing), all negative or within normal range. His C-reactive protein (CRP) and erythrocyte sedimentation rate (ESR) were elevated at 12.5 mg/dl and 89 mm/h, respectively, consistent with an inflammatory process (Table 1). Rheumatologic laboratories included negative rheumatoid factor (RF) and anti-cyclic citrullinated peptide (CCP), while the antinuclear antibodies (ANA) were positive with a titer of 1:160, but the rest of the extractable nuclear antigen (ENA) panel was negative. Thyroid-stimulating hormone (TSH) level was normal. He was treated with nonsteroidal anti-inflammatory drugs (NSAIDs) for possible acute inflammatory response with IA secondary to the Pfizer vaccine and discharged home.

**Table 1:** Clinical characteristics of patient 1 ANA: Antinuclear antibodies; ENA: Extractable nuclear antigen; CCP: Cyclic citrullinated peptide; ESR: Erythrocyte sedimentation rate; CRP: C-reactive protein; CPK: Creatine phosphokinase.

Labs	April 24, 2021	July 26, 2021	September 20, 2021
ANA	1:160 homogeneous	Negative	
ENA panel	Negative		
Rheumatoid factor (IU/ml)	Negative	11	
Anti-CCP (U/ml)	6	<1	
ESR (mm/h)	89	46	17
CRP (mg/dL)	12.5	10.8	2.36
CPK	47	35	
Extended myositis panel		Negative	
Hemoglobin (g/dl)		11.8	
Platelets		409,000	
Albumin (g/dl)		3.8	
Electromyography		Normal	

The patient initially responded to NSAIDs, but symptoms returned as they were stopped. Then, he was seen by his primary care physician (PCP) two weeks after his hospital discharge. At that time, the patient still had the same symptoms of generalized weakness and polyarthralgia. He was started on prednisone 20 mg orally daily for two weeks by his PCP with a good response and resolution of his symptoms. His symptoms flared again when off prednisone.

The patient was then seen by rheumatology a few weeks later as an outpatient consult for persistent symptoms of generalized weakness and diffuse joint pain. He complained of pain in all of his joints and morning stiffness that lasted a few minutes. He also reported bilateral hand swelling. On physical exam, he had bilateral hand swelling with tenderness and swelling of his right fourth and fifth metacarpophalangeal (MCP) joints, with no other active synovitis. Vaccine-associated new-onset IA was the working diagnosis. His labs including ESR and CRP were repeated. He was started on a longer tapering course of prednisone for two months.

The patient was seen in a follow-up visit two weeks after he completed his prednisone taper, and by then his symptoms had subsided. His inflammatory markers trended down with the last ESR level of 17 mm/h and CRP level of 2.36 mg/dl. The decision was made to observe him off steroids and immunosuppressive medications.

Based on the symptoms, physical exam, elevated inflammatory markers, and response to steroids, this case suggests IA which likely was triggered by the immune response to the Pfizer COVID-19 vaccine, though waned over time.

Case 2: COVID vaccine-associated new-onset rheumatoid arthritis

A 74-year-old man, with a history of obstructive sleep apnea, depression, idiopathic pulmonary fibrosis on nintedanib 150 mg twice daily, asbestos exposure, hypertension, and gout, presented to his primary care clinic for polyarthralgia. He had recently received two doses of the Pfizer-BioNTech SARS-CoV-2 mRNA vaccine. The first and second doses of the vaccine were administered in January 2021 and February 2021, respectively. Ten days after the first dose, the patient reported bilateral hip and shoulder pain, associated with 15 minutes of morning stiffness, as well as left thumb pain. He also developed a rash overlying a few joints (the left ankle, the right knee, and the right wrist). He reported that the rash was migratory, pruritic, and papular in quality. Dermatology was consulted in February before the second dose. He was diagnosed with a serum sickness-like reaction due to the COVID-19 vaccine and was treated with antihistamines and steroid creams for the symptoms of rash. He was also referred to an infectious disease specialist who recommended the administration of the second dose as he did not have an anaphylactic or severe reaction to the vaccine.

The patient reported the same joint pain after the second dose, so he presented to the rheumatology outpatient clinic in late February. On physical exam, he was noted to have tenderness to palpation of the first carpometacarpal (CMC) joint with a positive grind test and tenderness to palpation of the right acromioclavicular (AC) joint, with mildly limited range of motion. No rash was present at that time. A full rheumatologic workup was performed (Table 2).

**Table 2:** Clinical characteristics of patient 2 ANA: Antinuclear antibodies; ENA: Extractable nuclear antigen; CCP: Cyclic citrullinated peptide; ESR: Erythrocyte sedimentation rate; CRP: C-reactive protein; CPK: Creatine phosphokinase; LDH: Lactate dehydrogenase.

Labs	Feb 18, 2021	March 5, 2021	March 31, 2021	April 14, 2021	June 3, 2021	September 3, 2021
ANA	1:320 homogeneous					
ENA panel	Negative					
Rheumatoid factor (IU/ml)	24					74
Anti-CCP (U/ml)		<1.5				
ESR (mm/h)	6		14		4	32
CRP (mg/L)	15.4	27.2	55.9	10.5	5	35
C3 complement (mg/dl)		123				
C4 complement (mg/dl)		21				
Hemoglobin (g/dl)	15.8		14.6		14.7	14.9
Platelets (k/cmm)	236,000		253,000		208,000	239,00
Albumin (g/dl)			3.5	3.6		
CPK (U/L)			67			
LDH (U/L)			258			
Uric acid (mg/dl)	3.6					3.6

Pending the results, the patient’s pruritic rash resumed in early March, and weakness in his thigh and shoulder muscles was noted. He started using a cane to walk, fell multiple times around the house, could not bend over, had difficulty rising from a seated position, and had trouble raising his hands above his head. Pain and stiffness were worse in the morning and at night, but improved during the daytime and after taking ibuprofen. He presented with these symptoms to the emergency department and was given an intramuscular ketorolac injection, which relieved most of his symptoms.

On the same day, he contacted his rheumatology provider, with only a few labs available. His CRP was 15 mg/L, and RF was 24 IU/ml (low positive). He was initially diagnosed with PMR, without any symptoms or findings for giant cell arteritis (GCA). He was prescribed prednisone 20 mg orally daily with a slow taper.

Subsequently, he was seen in the clinic in late March (two weeks after starting the steroids). Physical exam revealed motor power of 5/5 in all limbs. He reported improvement on the 20-mg prednisone dose, but symptoms recurred when the dose was tapered down to 15 mg. He had more pain and stiffness lasting two hours in the morning, with motor weakness.

His prednisone dose was increased to 30 mg, pending labs. On the same day, he presented again to the same emergency department with a cough that persisted for five days, which had not improved with azithromycin. He was discharged on a levofloxacin course upon being diagnosed with mild pneumonitis.

He gradually started tapering his steroids after symptoms improved, and his CRP trended down. However, whenever he tried to taper the prednisone below 10 mg, his symptoms returned, and he resumed the 10-mg daily prednisone dose. In June, his symptoms resolved with an associated normalization of inflammatory markers, CRP 5 mg/L, and ESR 4 mm/h.

Over the next two months, the patient attempted to decrease his prednisone dose further as instructed by his provider, but symptoms recurred when the daily prednisone dose was tapered below 10 mg. In early September, he developed arthritis of the right wrist and multiple MCPs. He also began experiencing stiffness and pain in the morning that improve during the day and with NSAIDs. Being the primary caretaker of his household, he had significant difficulty with his daily living activities. Six months after his initial presentation, the repeat laboratory testing showed a highly positive RF of 74. Hence, the clinical assessment at that time changed to RA, and a steroid-sparing agent was started. Given his pulmonary history, methotrexate was not an option, so he was started on leflunomide after his TB Quantiferon® and hepatitis panel testing results were negative.

Case 3: COVID vaccine-associated flare of inflammatory arthritis

A 76-year-old man with a history of seropositive RA (RF positive), an interstitial lung disease with stable CT chest findings for a duration of five years, normal pulmonary function tests, and a former smoker presented with a recent history of mild COVID-19 infection on December 29, 2020, with symptoms of cough, fatigue, and loose stools that improved without hospitalization. His RA has been treated with methotrexate (MTX) 25 mg subcutaneously weekly, hydroxychloroquine 200 mg twice daily, and abatacept 1000 mg IV infusion every four weeks, and the disease was under good control, with disease activity score 28 (DAS-28)-CRP of 2.46 (consistent with remission) during his last abatacept infusion. He was instructed to hold his MTX for two weeks after each COVID-19 vaccine dose to be able to mount an optimal immune response (the American College of Rheumatology or ACR guidance about delaying abatacept was not published yet). He was confused about the guidance provided about windowing MTX and ended up holding it for three weeks after the first vaccine dose.

Upon presentation for his scheduled abatacept infusion, he reported that a few days after his first Pfizer-BioNTech mRNA SARS-CoV-2 vaccine in early February 2021, he experienced bilateral inflammatory shoulder pain with stiffness. During that visit, DAS-28-CRP was 4.16, consistent with moderate disease activity. He proceeded to receive the second dose of the vaccine five days after his abatacept infusion and stopped his MTX again for two weeks. At his next abatacept infusion, in March, he reported pain in the shoulders, interphalangeal (PIP) joints, MCP joints, and wrists, with swelling and morning stiffness. DAS-28-CRP was 4.52, consistent with moderate disease activity. So, a steroid taper was prescribed. After the steroid taper, there was an improvement in his symptoms.

After completion of the taper and during his abatacept infusion in April, he reported 75% improvement in the joint symptoms, but he still had morning stiffness for 15-20 minutes. DAS-28-CRP was 5.53, which was consistent with the high disease activity. During his abatacept infusion in May, his symptoms improved, but he had a rise in inflammatory markers with a DAS-28-CRP of 3.45, which was consistent with moderate disease activity. It was believed that his RA flare could be related to MTX windowing after each dose of the COVID-19 vaccine versus RA flare triggered by the COVID-19 vaccine itself versus wane in the effectiveness of abatacept. A decision was made to continue with the current regimen, adding a short course of prednisone, and to monitor his response to therapy after resuming his MTX.

In the subsequent three abatacept infusions (June, July, and August), he was clinically improved (85% improvement) with normal inflammatory markers. DAS-28-CRP of 2.64 indicates low disease activity; DAS-28-CRP of 2.78 indicates low disease activity, and DAS-28-CRP of 3.58 indicates moderate disease activity (see Table 3).

**Table 3:** Clinical markers of disease activity of patient 3 ESR: Erythrocyte sedimentation rate; DAS-28: Disease activity score 28; CRP: C-reactive protein.

Date	DAS-28-CRP	ESR (mm/h)	CRP (mg/L)	Hemoglobin (g/dl)	Platelets (K/cmm)	Albumin (g/dl)
January	2.46	18	15.9	13.3	157,000	3.2
February	4.16	34	11.8	13.7	144,000	3.5
March	4.52	23	8	13.6	142,000	3.5
April	5.53	24	29	13.3	142,000	3.2
May	3.45	34	26	13.6	163,000	3.3
June	2.64	19	7.7	13.3	135,000	3.4
July	2.78	29	11	13.8	148,000	3.5
August	3.58	30	7.9	12.9	135,000	3.5
September		25		13.9	162,000	3.7
October	1.8	5	1.8	13.6	177,000	3.7

During his abatacept infusion in September, he reported two to three flares of his RA in the prior three weeks, with wrist and shoulder pain as well as swelling and morning stiffness for two hours, worse than all his previous symptoms. He required prednisone doses three times at home to alleviate his pain. On physical exam, he had a new rash. His arthritis was not controlled at that time, so his therapy was switched to tofacitinib 5 mg twice daily.

Dermatology was consulted for evaluation of the rash. It started in the feet and spread to the legs, trunk, gluteal region, and arms, sparing the face, palms, and soles. Physical exam showed erythematous macules coalescing into patches, with overlying adherent white scales. Reticular erythema over the thighs and back as well as pink plaques with scales on the penis and gluteal cleft were present. Ill-defined erythema and erosions in a V-shaped distribution over the neck were also noted. The differential diagnosis as per dermatology was psoriasis versus dermatomyositis. Two punch biopsies were performed by the dermatologist: one from the left mid-back region and another from the left shoulder.

Pathology Results

Left mid back: Spongiotic and interface dermatitis, likely drug eruption versus viral exanthema.

Left shoulder: Psoriasiform and spongiotic dermatitis, which can be seen in drug eruption, viral exanthema, or allergic contact dermatitis. Though psoriasis is less likely, erythrodermic psoriasis cannot be excluded.

The patient was started on tofacitinib in September. He presented to the clinic for a follow-up in late October. He denied any joint pains, swelling, or morning stiffness. DAS-28-CRP of 1.87 indicated remission of the disease.

Previous Immunosuppression

Infliximab: May 2008 to November 2008, which was stopped due to loss of effectiveness.

Adalimumab: April 2009 to July 2009, which was stopped due to ineffectiveness.

Rituximab: September 2009 to May 2010, which was stopped due to ineffectiveness.

Abatacept: August 2010 to September 2021, 1000 mg IV every four weeks, which was stopped due to loss of effectiveness.

## Discussion

In our case series, we discuss a case of a flare of RA over the course of the COVID vaccine series and two cases of new-onset IA, one temporary and one long-standing, requiring long-term medication management.

IA, with RA as a subset, can be categorized into many groups that cause joint pain, swelling, tenderness, and morning stiffness [2]. Arthritis can be either isolated or a part of a systemic disease with other manifestations. IA can be caused by various etiologies including infectious or noninfectious. Examples of noninfectious IA are crystal-induced arthropathy, RA, seronegative spondyloarthropathies, and arthritis associated with connective tissue diseases. Some fall into undifferentiated IA at the beginning, which could eventually develop into a well-defined systemic disease like RA, but some will remain undifferentiated IA.

In IA such as RA [3], various cytokines such as IL-1 (interleukin), IL-6, IL-8, and tumor necrosis factor-alpha (TNF-alpha) contribute to inflammation and autoimmunity leading to the destruction of joints. Macrophages and lymphocytes produce pro-inflammatory cytokines and chemokines such as TNF and granulocyte-macrophage colony-stimulating factor (GM-CSF) in the synovium. Dendritic cells present antigens to T-cells that are present in the synovium. Activation of the T-cells requires two signals. The first is antigen presentation to the T-cell receptor, and the second one is costimulatory signaling between cluster of differentiation (CD80-86) on the dendritic cells and CD28 on T-cells. When the latter become activated, they begin to proliferate and secrete additional cytokines including IL-2, interferon-gamma (IFN-γ), TNF, and IL-4. It is under the effect of these T-cell-derived cytokines that additional cells become activated. B-cells become activated through interactions with T-cells and differentiate into antibody-forming plasma cells. These inflammatory cells mediate an immune response in genetically susceptible individuals to cause RA.

In our first case, the patient developed IA after he received his COVID vaccines. He had a good clinical response with steroids but flared after discontinuing the two-week course. It was evident that he required a long-term steroid taper, which eventually helped resolve his arthritis. No steroid-sparing agent was required; then, the steroids were stopped eventually after only a few months, and the symptoms resolved. This shows that a temporary IA can occur after COVID vaccination, requiring a short course of steroids only without the need for conventional disease-modifying rheumatic drugs (cDMARDs), with a good clinical outcome.

PMR is an auto-inflammatory condition characterized clinically by aching/pain in the shoulders, hip girdle, and neck associated with morning stiffness that can last from 45 minutes to hours, with the gelling phenomenon. Systemic manifestations such as malaise, fever, depression, weight loss, and anorexia may be present. It can be associated with GCA in 5%-30% of cases; the two disorders may represent different manifestations of a shared disease process [4]. The cause of PMR is not known; some speculate environmental infectious triggers such as viruses are involved, but studies have been inconsistent.

Laboratory findings lean toward a predominant role of innate immune response in PMR. In circulating CD4+ T-cell subsets, T helper 17 (Th17) cells are increased, and regulatory T (Treg) cells are decreased. The pro-inflammatory cytokine IL-6 is elevated in the peripheral blood of patients with PMR and is thought to be responsible for their constitutional manifestations as well as elevated inflammatory markers such as ESR and CRP. Further pathobiological findings supporting the key role of innate immunity in PMR pathogenesis are represented by the increased expression of toll-like receptor TLR-7 and TLR-9 in peripheral blood monocytic cells and the emerging involvement of Th17 cells [5].

In our second case, the patient initially presented with symptoms suggestive of PMR post-vaccine, with mainly hip and shoulder girdle pains, stiffness, and negative serology for autoimmune disease. He responded well initially to steroids but relapsed with doses lower than 10 mg of prednisone. A few months after his initial presentation, the patient started developing peripheral arthritis suggestive more of RA. Subsequent laboratory workup revealed a significant rise in his RF, and no CCP was available at the time. The patient slowly transformed from IA-like PMR picture to a long-term autoimmune disease suggestive of seropositive RA. He eventually required a cDMARD, leflunomide, to control his arthritis with a good clinical response.

Vaccines and viruses have been implicated in the triggering theory of auto-inflammatory/autoimmune diseases with multiple studies documenting it. This new mRNA COVID vaccine has been shown to elicit TH1 cell responses after the first dose, with 0.05% of circulating CD4+ T-cells secreting TNF and/or IL-2 following in vitro stimulation with S protein peptides [6]. It can also stimulate the innate immunity through endosolic and cytoplasmic nucleic acid receptors, such as toll-like receptors (TLRs), especially TLR-7 and TLR-9. Stimulation of these receptors will lead to the transcription and translation of multiple inflammatory molecules, such as IL-6, interferon-alpha (IFN-alpha), TNF-alpha, and IL-27. These cytokines are responsible for a myriad of inflammatory conditions, including RA, where these molecules lead to synovial inflammation and joint destruction. It has also been documented that the peripheral mononuclear blood cells of patients with PMR have an increased expression of TLR-7 and TLR-9. The stimulation of these receptors as well as the downstream stimulation of the aforementioned inflammatory cytokines could be one of the possible theories or links to how PMR or RA/IA can clinically manifest a few days post vaccine administration.

New cases of auto-inflammatory or autoimmune diseases as well as flares have been documented to occur post influenza; measles, mumps, rubella (MMR); hepatitis, and diphtheria, tetanus, pertussis (DTP) vaccines, including PMR and RA [7]. An analysis of over 500 cases of these diseases was published in several countries, such as UK, Italy, Israel, Spain, Portugal, and Russia [8]. It reported four cases of PMR and 10 cases of RA post influenza vaccine. The theory linking the vaccine to these cases is autoimmune/inflammatory syndrome induced by adjuvants (ASIA) also known as Shoenfeld's syndrome, which has been reported by many scientists with over 4400 documented cases worldwide, but it has also been refuted by others. It appears that adjuvants and compounds with adjuvant properties in the new mRNA vaccines (like aluminum salt-based adjuvants or liquid paraffin, silicone gel, acrylamides, hyaluronic acid, methacrylate, and others), similar to influenza and other vaccines, can induce these diseases [9]. Our patients fulfill two major criteria of the syndrome; for the diagnosis of ASIA, there must be at least two major criteria or one major and two minor criteria present [10].

Suggested criteria for the diagnosis of "ASIA"

Major Criteria

The major criteria include (1) exposure to an external stimulus (infection, vaccine, silicone, adjuvant) prior to clinical manifestations; (2) the appearance of "typical" clinical manifestations such as myalgia, myositis, or muscle weakness; arthralgia and/or arthritis; chronic fatigue, un-refreshing sleep, or sleep disturbances; neurological manifestations (especially associated with demyelination); cognitive impairment and memory loss; pyrexia and dry mouth; (3) removal of inciting agent that induces improvement; and (4) typical biopsy of involved organs.

Minor Criteria

The minor criteria include (1) the appearance of autoantibodies or antibodies directed at the suspected adjuvant; (2) other clinical manifestations, i.e., irritable bowel syndrome; (3) specific human leukocyte antigen (HLA), i.e., HLA DRB1, HLA DQB1; and (4) evolvement of an autoimmune disease, i.e., multiple sclerosis and systemic sclerosis.

Given that the COVID vaccines, mRNA, and others have only been available to the public for a year and a half now, documented post-vaccine cases of new or flares of existing autoimmune/auto-inflammatory cases have been scarce. In our literature review, we came across one case report of a new PMR case following COVID-19 vaccine administration, with symptoms developing just a few days after the injection, and clinical improvement after a short steroid course [11]. A multicenter study reported 27 cases of immune-mediated disease flares or new-onset disease in subjects following mRNA/DNA SARS-CoV-2 vaccination from the USA, Canada, the UK, and Israel [12]. It reported one case of PMR flare seven days after the first dose, one case of new-onset PMR three days after the first dose, and five cases of RA flare (one occurred seven days after the second dose, one on the third day after the second dose, one after four days of the second dose, one after 25 days, and the last one after two days of the first dose).

In a study conducted at our center, 60 RA patients who were on active immunosuppressant medications were contacted by phone after they take their Pfizer COVID-19 vaccines to evaluate the development of adverse events or RA flares [13]. Out of all the patients, only one reported an RA flare following the vaccine, and he already had active disease prior to taking it. The rest reported mainly soreness over the injection site, fatigue, headache, and arthralgia for a few days that resolved spontaneously. No major side effects were reported.

In our third and last case, the patient already had an established autoimmune disease, which is RA. After the patient had his COVID vaccines, his previously well-controlled RA on abatacept worsened, and he started requiring steroid therapy on multiple occasions to control his flares. Eventually, his symptoms persisted, and his inflammatory markers remained elevated necessitating a switch to another agent, due to secondary failure of abatacept. He was switched to tofacitinib with subsequent follow-ups showing remission of his disease. The clinical picture of this case is more complex as the reason that actually caused his loss of response to abatacept is not obvious. Three speculations arise here: (1) Is it vaccine-related due to the timing of the shots and the worsening of his arthritis? (2) Is it related to the MTX windowing (although this does not explain the persistent arthritis with resuming MTX for months after the vaccine)? Or is it related to the abatacept with a secondary failure occurring around the time of the vaccine, coincidentally? It is difficult to discern what exactly caused this outcome with abatacept, making it crucial to consider all possibilities contributing to this situation and necessitating the need to tailor therapy or its modification on an individualized patient level.

Given the timeline of when our patients developed either a flare of their existing RA or new-onset IA or PMR (a few days after receiving the COVID-19 vaccine), in addition to the currently available evidence of documented similar cases post administration of mRNA vaccines, as well as the link between their mechanism of action and the pathogenesis of those diseases, we can speculate a causal relationship between the vaccine and the triggering of these disease entities. The strength of our case series is that multiple patients with or without a prior rheumatologic diagnosis had an adverse immune-related reaction, similar to other worldwide reports as well as a study from our center. The limitation of our study is the size of our series, which is only three patients.

## Conclusions

These three cases highlight three considerations that need to be taken into account while managing our autoimmune disease population during the COVID vaccine series and evaluating new-onset IA. First, each patient with autoimmune disease is at theoretical (low incidence) risk of the flare of disease due to either the immune-activating aspects of the vaccine itself, the windowing of immune-suppressive medications used to control the vaccine in an attempt to optimize vaccine response, or the coinciding natural course of autoimmune disease that waxes and wanes. The long-term management in the third case was not negatively impacted, so even though a flare in autoimmune disease may have been in part attributable to the vaccine, the risk of vaccine exposure was not a long-term complication. The second is that new-onset temporary inflammatory disease is a potential non-life-threatening complication of administering immunogenic vaccines. This has been known for some time in the context of many vaccines and appears to be associated with the COVID vaccine as well. The third consideration is that there are many environmental triggers of autoimmune disease, and vaccine administration is one of them that rarely may include COVID-19 vaccines. Further evaluation of the incidence of the latter will provide additional clarity, though the rarity of this occurrence in the setting of over half the US population becoming vaccinated indicates that the benefit of the vaccine in terms of protection from COVID morbidity and mortality far outweighs the risk of this occurrence.
